# Exercise-responsive endocrine-metabolic mediators in diabetes and cardiometabolic disorders: a systematic scoping review and evidence map

**DOI:** 10.3389/fendo.2026.1892959

**Published:** 2026-09-22

**Authors:** Zihang Zhao, Yousheng Zhang, Zihan Liu, Qianye Li, Teng Pan, Shuo Yang, Xin Wang, Lianxin Song, Zhuoyi Li, Ruipeng Zhang, Zhiyong Hou

**Affiliations:** 1 Department of Orthopaedic Surgery, Third Hospital of Hebei Medical University, Shijiazhuang, China; 2 Hebei Medical University, Shijiazhuang, China; 3 Faculty of Medicine, Dentistry and Health Sciences, The University of Melbourne, Melbourne, VIC, Australia; 4 Department of Oncology, Shijiazhuang People’s Hospital, Shijiazhuang, China

**Keywords:** adipokines, cardiometabolic disorders, diabetes, evidence map, exercise, exerkines, extracellular vesicles, myokines

## Abstract

**Background:**

Exercise-responsive endocrine-metabolic mediators may contribute to the systemic cardiometabolic adaptations induced by physical activity, but the literature spans diverse mediator classes, exercise paradigms, disease contexts, and experimental models. We conducted a systematic scoping review and evidence map to characterize the structure of this evidence and the biological relations examined across the field.

**Methods:**

PubMed/MEDLINE, Web of Science Core Collection, and Scopus were searched from database inception through 7 August 2026. Titles and abstracts, followed by full texts, were independently assessed by two reviewers, with third-reviewer adjudication where required. Eligible primary reports underwent a separate round of independent full-text data charting using a relational framework linking reports, exercise contrasts, and mediator evidence. Cardiometabolic contexts and outcome domains were coded using multi-label rules, synonymous mediator terminology was harmonized, and companion publications were linked before study-level synthesis. Six potentially co-occurring evidence relations were mapped: exercise responsiveness, cardiometabolic association, source/secretion evidence, receptor/pathway evidence, functional perturbation, and formal mediation.

**Results:**

The searches yielded 3,388 records, of which 1,535 unique records underwent title/abstract screening and 397 reports underwent full-text assessment. The final primary evidence map comprised 365 reports representing 363 underlying studies, 413 exercise contrasts, 776 mediator-evidence observations, and 103 harmonized named mediators. Obesity/overweight was represented in 78.0% of studies. Among the 308 studies contributing named mediators, adipokines were represented in 58.4%, cytokines/chemokines in 32.1%, and myokines in 25.6%. Adiponectin, leptin, IL-6, and irisin were the most frequently represented named mediators. Exercise-responsive evidence and cardiometabolic associations were identified in 90.9% and 87.0% of contributing studies, respectively, whereas source/secretion evidence (18.5%), receptor/pathway evidence (9.7%), functional perturbation (11.4%), and formal mediation (0.3%) were less frequently represented. Chronic training accounted for 81.6% of exercise contrasts and aerobic/endurance exercise for 63.4%.

**Conclusions:**

Exercise-responsive mediator research in cardiometabolic disease comprises a concentrated group of recurrent adipokines, cytokines, and myokines alongside a broad, sparsely studied mediator landscape. Exercise-responsive evidence and cardiometabolic associations were frequently represented, whereas direct investigation of tissue source, downstream pathways, functional perturbation, and formal mediation was less common. Studies integrating well-characterized exercise exposures with serial mediator assessment, source validation, and mechanistic testing may help define how exercise-responsive signaling contributes to cardiometabolic adaptation.

## 1 Introduction

Exercise is an integral component of the prevention and management of diabetes and related cardiometabolic disorders (1). Its benefits extend across glycemic regulation, insulin sensitivity, body composition, cardiorespiratory fitness, vascular function, and inflammatory homeostasis, reflecting coordinated adaptations that involve multiple organs rather than skeletal muscle alone (2–4). This systemic response has increasingly shifted attention toward the molecular signals that accompany exercise and may participate in communication among skeletal muscle, adipose tissue, liver, the cardiovascular system, and other tissues.

An important foundation for this field came from human studies showing that contracting skeletal muscle releases interleukin-6 into the circulation during exercise (5), followed by the development of the myokine concept to describe muscle-derived signaling molecules with local or systemic actions (6). Subsequent work broadened this perspective to exerkines, encompassing signaling factors released or mobilized in response to acute exercise or exercise training from multiple tissues (3, 4, 7). These terms, however, describe different biological attributes. Source-oriented terms such as myokine and adipokine refer to an attributed tissue origin, whereas exercise responsiveness refers to the context in which a mediator is observed or released. Moreover, many circulating factors can arise from more than one tissue and can have context-dependent actions (3, 7). An exercise-associated change in circulating concentration therefore does not, on its own, establish tissue origin, active secretion, downstream receptor or pathway involvement, or a functional role in the resulting cardiometabolic response. For this reason, we use exercise-responsive endocrine-metabolic mediators as a broad working term and consider evidence of exercise responsiveness, biological source, and downstream action separately.

The scale of discovery has also expanded considerably. Proteomic characterization of exercise-responsive extracellular vesicles demonstrated a potential route for inter-tissue transfer of molecular cargo (8), while longitudinal multi-omic profiling revealed extensive and temporally coordinated metabolic, inflammatory, cardiovascular, and tissue-repair responses to an acute exercise bout (9). More recently, the Molecular Transducers of Physical Activity Consortium mapped molecular adaptations across multiple tissues and omic layers during endurance training (10). These advances have enlarged the candidate mediator landscape but have also made interpretation more demanding. Acute responses cannot necessarily be extrapolated to chronic training adaptations, and the timing of post-training sampling relative to the most recent exercise bout can materially affect the observed molecular profile (11). Likewise, evidence that a mediator changes with exercise, correlates with a cardiometabolic phenotype, originates from a particular tissue, engages a receptor or signaling pathway, produces an effect under experimental perturbation, or statistically mediates an exercise–outcome relationship addresses distinct questions. Preserving these distinctions is important when organizing a rapidly expanding literature.

Existing systematic syntheses illustrate both the progress of the field and the specificity of the questions examined to date. In type 2 diabetes, García-Hermoso et al. synthesized randomized trials evaluating training-related changes in a predefined panel of exerkines and their relationships with metabolic outcomes (12). Bettariga et al. examined the acute responses of selected myokines to different exercise modes in healthy adults (13). Other meta-analyses have focused specifically on immunoregulatory myokines following acute resistance exercise (14) or acute endurance exercise (15). Together with broader conceptual reviews of exerkines and cardiometabolic health (3, 4, 7), these studies have clarified important mediator-, population-, and exercise-specific patterns. A complementary question is how the underlying primary literature as a whole is distributed across cardiometabolic contexts, exercise exposures, mediator identities, biological models, and the different forms of evidence used to connect exercise-responsive signals with downstream biology.

We therefore conducted a systematic scoping review and evidence map of exercise-responsive endocrine-metabolic mediators in diabetes and cardiometabolic disorders. We aimed to characterize the primary evidence across cardiometabolic contexts, exercise characteristics, mediator families and named mediators, and human and experimental models. To distinguish the biological questions addressed by different studies, evidence was mapped separately for exercise-responsive change, cardiometabolic association, source or secretion, receptor or pathway involvement, functional perturbation, and formal mediation. This framework provides a structured view of where research has accumulated and which aspects of exercise-responsive signaling have been examined through more direct experimental or mediational approaches.

## 2 Materials and methods

### 2.1 Review design and reporting

We conducted a systematic scoping review and evidence map to characterize the primary literature on exercise-responsive endocrine-metabolic mediators in diabetes and cardiometabolic disorders. The review was informed by updated JBI methodological guidance for scoping reviews and reported in accordance with the PRISMA Extension for Scoping Reviews (PRISMA-ScR); reporting of the literature search was additionally guided by PRISMA-S (16–18). The review was designed to map the distribution and characteristics of the available evidence rather than to estimate a pooled intervention effect. No protocol was prospectively registered.

The review question and eligibility framework were structured according to Population–Concept–Context principles. The analytical framework distinguished reports, underlying studies, exercise contrasts, and mediator-level evidence so that each summary could be calculated using the denominator appropriate to the corresponding research question.

### 2.2 Information sources and search strategy

PubMed/MEDLINE, Web of Science Core Collection (WoSCC), and Scopus were searched from database inception through 7 August 2026, with all final searches conducted on 7 August 2026. No publication-date restriction was applied; reports from any publication year were eligible for consideration through the final search date. The final search strategy used the same three conceptual components across databases: an exercise-responsive mediator block, an inclusive cardiometabolic-context block, and a broad molecular/biological relevance block incorporating mechanism-, biomarker-, signaling-, and omics-related terminology together with terms for inflammation, oxidative stress, mitochondrial biology, and endothelial function. Metabolic and cardiovascular terms were combined within a single inclusive context block and were not required to co-occur. No language, publication-type, document-type, or journal-source filters were applied at the database-query level; eligibility restrictions were applied during screening.

PubMed combined Medical Subject Headings with title/abstract terms, whereas WoSCC used Topic fields and Scopus used TITLE-ABS-KEY. The final searches retrieved 898 records from PubMed/MEDLINE, 1,099 from WoSCC, and 1,391 from Scopus.

PubMed/MEDLINE

(

exerkine*[tiab]

OR “exercise-induced factor”[tiab]

OR “exercise-induced factors”[tiab]

OR “exercise-responsive factor”[tiab]

OR “exercise-responsive factors”[tiab]

OR “exercise-stimulated factor”[tiab]

OR “exercise-stimulated factors”[tiab]

OR “exercise-induced molecule”[tiab]

OR “exercise-induced molecules”[tiab]

OR “exercise-responsive molecule”[tiab]

OR “exercise-responsive molecules”[tiab]

OR “exercise-stimulated molecule”[tiab]

OR “exercise-stimulated molecules”[tiab]

OR (

(

myokine*[tiab]

OR adipokine*[tiab]

OR hepatokine*[tiab]

OR cardiokine*[tiab]

OR osteokine*[tiab]

OR organokine*[tiab]

OR secretome[tiab]

OR “extracellular vesicle”[tiab]

OR “extracellular vesicles”[tiab]

OR exosome*[tiab]

)

AND

(

“Exercise”[Mesh]

OR “Exercise Therapy”[Mesh]

OR “Motor Activity”[Mesh]

OR exercise*[tiab]

OR “physical activity”[tiab]

OR “physical activities”[tiab]

OR “exercise training”[tiab]

OR “aerobic exercise”[tiab]

OR “aerobic training”[tiab]

OR “endurance exercise”[tiab]

OR “endurance training”[tiab]

OR “resistance exercise”[tiab]

OR “resistance training”[tiab]

)

)

)

AND

(

“Diabetes Mellitus”[Mesh]

OR “Diabetes Mellitus, Type 1”[Mesh]

OR “Diabetes Mellitus, Type 2”[Mesh]

OR “Prediabetic State”[Mesh]

OR “Insulin Resistance”[Mesh]

OR “Obesity”[Mesh]

OR “Metabolic Syndrome”[Mesh]

OR “Cardiovascular Diseases”[Mesh]

OR “Endothelial Dysfunction”[Mesh]

OR “Atherosclerosis”[Mesh]

OR “Coronary Artery Disease”[Mesh]

OR “Heart Failure”[Mesh]

OR diabet*[tiab]

OR “type 1 diabetes”[tiab]

OR “type 2 diabetes”[tiab]

OR T1D[tiab]

OR T2D[tiab]

OR prediabet*[tiab]

OR “insulin resistance”[tiab]

OR hyperglycemi*[tiab]

OR obes*[tiab]

OR overweight[tiab]

OR “metabolic syndrome”[tiab]

OR “glucose metabolism”[tiab]

OR cardiometabolic[tiab]

OR “cardio-metabolic”[tiab]

OR MASLD[tiab]

OR NAFLD[tiab]

OR “metabolic dysfunction-associated steatotic liver disease”[tiab]

OR “nonalcoholic fatty liver disease”[tiab]

OR “non-alcoholic fatty liver disease”[tiab]

OR “cardiovascular disease”[tiab]

OR “cardiovascular diseases”[tiab]

OR “endothelial dysfunction”[tiab]

OR “endothelial function”[tiab]

OR “vascular dysfunction”[tiab]

OR “vascular function”[tiab]

OR atherosclero*[tiab]

OR “coronary artery disease”[tiab]

OR “coronary heart disease”[tiab]

OR “heart failure”[tiab]

OR “diabetic cardiomyopathy”[tiab]

OR “diabetic cardiomyopathies”[tiab]

OR “cardiac remodeling”[tiab]

OR “cardiac remodelling”[tiab]

OR “myocardial dysfunction”[tiab]

)

AND

(

mechanism*[tiab]

OR signaling[tiab]

OR signalling[tiab]

OR pathway*[tiab]

OR biomarker*[tiab]

OR omics[tiab]

OR proteomic*[tiab]

OR metabolomic*[tiab]

OR transcriptomic*[tiab]

OR “interorgan crosstalk”[tiab]

OR “organ crosstalk”[tiab]

OR mitochondria*[tiab]

OR mitochondrial*[tiab]

OR inflammation[tiab]

OR inflammatory[tiab]

OR “oxidative stress”[tiab]

OR “endothelial function”[tiab]

OR “endothelial dysfunction”[tiab]

)

Web of Science Core Collection

(

TS=(exerkine* OR “exercise-induced factor*” OR “exercise-responsive factor*”

OR “exercise-stimulated factor*” OR “exercise-induced molecule*”

OR “exercise-responsive molecule*” OR “exercise-stimulated molecule*”)

OR (

TS=(myokine* OR adipokine* OR hepatokine* OR cardiokine* OR osteokine*

OR organokine* OR secretome OR “extracellular vesicle*” OR exosome*)

AND TS=(exercise* OR “physical activit*” OR “exercise training”

OR “aerobic exercise” OR “aerobic training” OR “endurance exercise”

OR “endurance training” OR “resistance exercise” OR “resistance training”)

)

)

AND TS=(diabet* OR “type 1 diabetes” OR “type 2 diabetes” OR T1D OR T2D

OR prediabet* OR “insulin resistance” OR hyperglycemi* OR obes* OR overweight

OR “metabolic syndrome” OR “glucose metabolism” OR cardiometabolic

OR “cardio-metabolic” OR MASLD OR NAFLD

OR “metabolic dysfunction-associated steatotic liver disease”

OR “nonalcoholic fatty liver disease” OR “non-alcoholic fatty liver disease”

OR “cardiovascular disease*” OR “endothelial dysfunction” OR “endothelial function”

OR “vascular dysfunction” OR “vascular function” OR atherosclero*

OR “coronary artery disease” OR “coronary heart disease” OR “heart failure”

OR “diabetic cardiomyopath*” OR “cardiac remodeling” OR “cardiac remodelling”

OR “myocardial dysfunction”)

AND TS=(mechanism* OR signaling OR signalling OR pathway* OR biomarker* OR omics

OR proteomic* OR metabolomic* OR transcriptomic* OR “interorgan crosstalk”

OR “organ crosstalk” OR mitochondria* OR mitochondrial* OR inflammation

OR inflammatory OR “oxidative stress” OR “endothelial function”

OR “endothelial dysfunction”)

Scopus

(

TITLE-ABS-KEY(exerkine* OR “exercise-induced factor*” OR “exercise-responsive factor*”

OR “exercise-stimulated factor*” OR “exercise-induced molecule*”

OR “exercise-responsive molecule*” OR “exercise-stimulated molecule*”)

OR (

TITLE-ABS-KEY(myokine* OR adipokine* OR hepatokine* OR cardiokine* OR osteokine*

OR organokine* OR secretome OR “extracellular vesicle*” OR exosome*)

AND TITLE-ABS-KEY(exercise* OR “physical activit*” OR “exercise training”

OR “aerobic exercise” OR “aerobic training” OR “endurance exercise”

OR “endurance training” OR “resistance exercise” OR “resistance training”)

)

)

AND TITLE-ABS-KEY(diabet* OR “type 1 diabetes” OR “type 2 diabetes”

OR T1D OR T2D OR prediabet* OR “insulin resistance” OR hyperglycemi*

OR obes* OR overweight OR “metabolic syndrome” OR “glucose metabolism”

OR cardiometabolic OR “cardio-metabolic” OR MASLD OR NAFLD

OR “metabolic dysfunction-associated steatotic liver disease”

OR “nonalcoholic fatty liver disease” OR “non-alcoholic fatty liver disease”

OR “cardiovascular disease*” OR “endothelial dysfunction” OR “endothelial function”

OR “vascular dysfunction” OR “vascular function” OR atherosclero*

OR “coronary artery disease” OR “coronary heart disease” OR “heart failure”

OR “diabetic cardiomyopath*” OR “cardiac remodeling” OR “cardiac remodelling”

OR “myocardial dysfunction”)

AND TITLE-ABS-KEY(mechanism* OR signaling OR signalling OR pathway*

OR biomarker* OR omics OR proteomic* OR metabolomic* OR transcriptomic*

OR “interorgan crosstalk” OR “organ crosstalk” OR mitochondria*

OR mitochondrial* OR inflammation OR inflammatory OR “oxidative stress”

OR “endothelial function” OR “endothelial dysfunction”)

Full execution details and the same database-specific search strategies are additionally provided in **Supplementary Table S1**.

### 2.3 Eligibility criteria

Eligible evidence included original studies in humans, animals, or cell/ex vivo systems that evaluated an eligible exercise or physical-activity exposure together with a circulating, secreted, extracellular-vesicle-associated, or other intercellular mediator relevant to diabetes or cardiometabolic biology. Human studies could involve diabetes, obesity/overweight, insulin resistance or prediabetes, metabolic syndrome, metabolic dysfunction-associated steatotic liver disease/non-alcoholic fatty liver disease (MASLD/NAFLD), cardiovascular disease, endothelial or vascular phenotypes, or related cardiometabolic states. Studies in otherwise healthy populations or experimental models were also eligible when they directly addressed a cardiometabolic phenotype or pathway relevant to the review question.

Eligible exposures included acute exercise, chronic exercise training, habitual physical activity, and experimental muscle-contraction paradigms. Exercise responsiveness and tissue origin were treated as separate attributes. Thus, the use of a source-oriented term such as myokine, adipokine, or hepatokine was not by itself considered evidence that a study had established tissue origin or active secretion.

The primary evidence map was restricted to original research for which an English-language full text was available. Reviews and other non-primary sources that remained useful for interpretation were retained separately as contextual sources but did not contribute to the primary analytic denominators.

### 2.4 Record management and study selection

Records retrieved from the three databases were combined and deduplicated sequentially using normalized digital object identifier (DOI) and PubMed identifier (PMID) matching, followed by manual adjudication of residual exact-title duplicate candidates. The deduplicated dataset was used for study selection.

**Figure 1:**
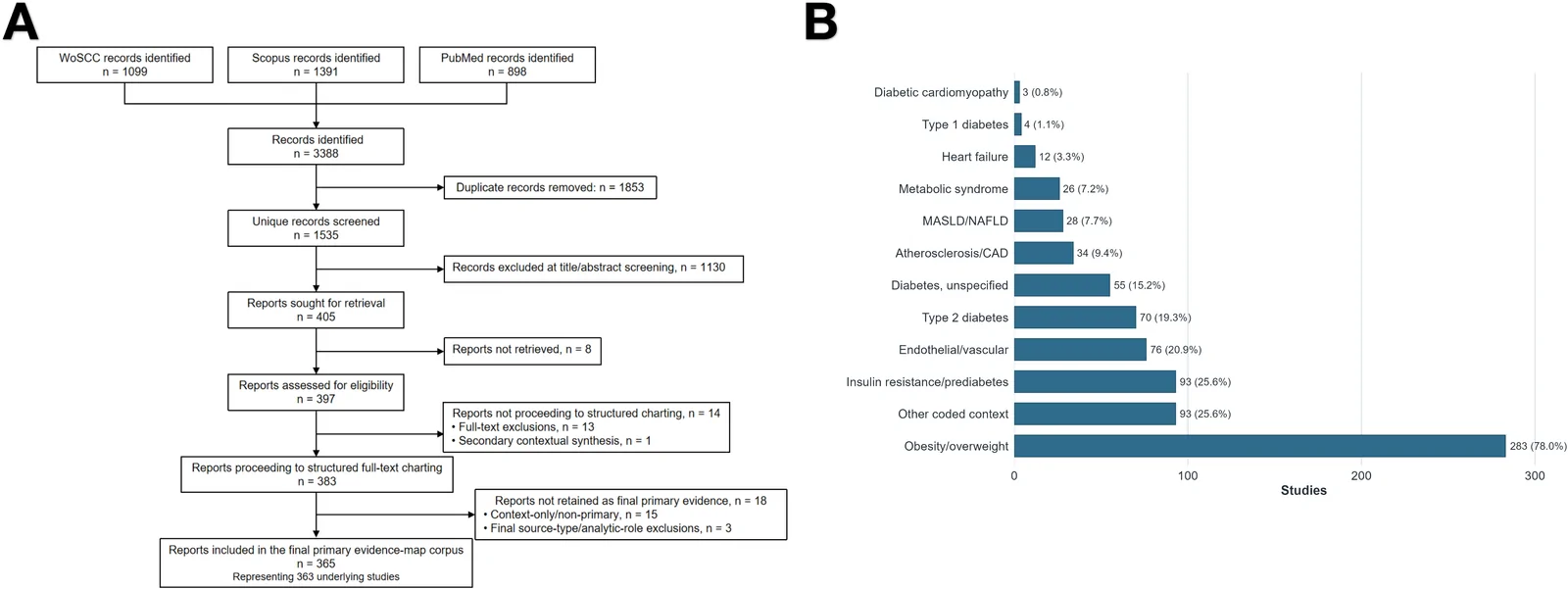
Study selection and cardiometabolic context coverage. **(A)** Study-selection flow for the systematic scoping review and evidence map. The three database searches identified 3,388 records. After sequential identifier-based deduplication and manual resolution of residual exact-title duplicates, 1,535 unique records underwent title/abstract screening. A total of 405 reports were sought for full-text retrieval; eight could not be retrieved, leaving 397 reports for full-text assessment. Thirteen reports were excluded at full-text assessment and one secondary contextual synthesis was retained outside the primary evidence stream, leaving 383 reports that proceeded to structured full-text charting. Following source-type and analytic-role adjudication during charting, 15 reports were retained as context-only/non-primary evidence and three were not retained in the final primary analytic dataset. The final primary evidence-map corpus comprised 365 reports representing 363 underlying studies. **(B)** Distribution of cardiometabolic contexts across the 363 underlying studies. Contexts were coded as non-mutually exclusive categories; individual studies could therefore contribute to more than one context and percentages need not sum to 100%.

Title and abstract screening was performed independently by two reviewers using the prespecified eligibility criteria. Potentially eligible reports then underwent independent full-text assessment by two reviewers. Disagreements at both stages were resolved by a third reviewer. Reasons for exclusion were recorded at full-text assessment, and the final study-selection process is presented in **Figure 1A**.

Reports arising from the same underlying study were subsequently linked to prevent companion publications from being counted as independent studies in study-level summaries. Report-level information was nevertheless retained for bibliographic traceability.

### 2.5 Data charting and adjudication

Data charting was organized at three linked levels: report, exercise contrast, and mediator evidence. Report-level variables included bibliographic information, study design, population or experimental model, and cardiometabolic context. Exercise-contrast variables captured temporality, modality, intensity, comparator structure, and other available prescription characteristics. Mediator-level variables included the reported mediator, harmonized mediator identity and family, biological model, biospecimen or tissue context, sampling time, direction of change, cardiometabolic outcome domains, and evidence-relation indicators.

The initial full-corpus charting exercise was treated as diagnostic and was used to identify ambiguities in the operational rules. A revised 20-report pilot was then used to refine the final charting framework. After these refinements, both reviewers independently re-charted all 383 reports that proceeded to structured charting *de novo* using new blank workbooks. Earlier diagnostic charting was not carried forward as the final extraction dataset. Differences in report structure, exercise contrasts, mediator identification, and field-level coding were resolved using prespecified reconciliation rules and third-reviewer adjudication where required.

Original study terminology was retained for traceability, while centrally harmonized variables were used for synthesis. Cardiometabolic contexts and outcome domains were coded as non-mutually exclusive multi-label variables. Named mediators were normalized to preferred labels and assigned to harmonized mediator families. The charting framework, controlled vocabulary, and operational definitions relevant to the reported analyses are provided in **Supplementary Table S2**, and the harmonized named-mediator vocabulary is provided in **Supplementary Table S4**.

### 2.6 Evidence-relation classification

Mediator evidence was mapped using six distinct, potentially co-occurring relations. Exercise-responsive evidence indicated that a mediator changed in relation to exercise, physical activity, or experimental contraction. Cardiometabolic association indicated an association between the mediator and a relevant metabolic, vascular, or cardiac phenotype. Source/secretion evidence required direct investigation of tissue origin, production, release, or secretion. Receptor/pathway evidence required direct investigation of a receptor or downstream molecular pathway involving the mediator. Functional perturbation evidence included experimental manipulation such as knockout, knockdown, blockade, supplementation, transfer, or an analogous intervention used to test mediator function. Formal mediation required an explicit mediation or indirect-effect analysis testing whether a mediator statistically linked an exercise-related exposure to a downstream outcome.

These relations were treated as parallel descriptors rather than as an ordinal hierarchy of evidence strength, and individual studies could contribute to more than one relation. Detailed operational definitions are provided in **Supplementary Table S2**.

### 2.7 Evidence synthesis

The synthesis was descriptive and denominator-specific. Reports were retained for publication-level traceability, whereas linked underlying studies were used for duplication-safe study-frequency summaries. Exercise characteristics were summarized at the exercise-contrast level. Named-mediator coverage was summarized using unique study×mediator units, and each underlying study contributed at most once to a given mediator-family or mapped evidence cell.

Because cardiometabolic contexts, outcome domains, biological models, and evidence relations could co-occur, percentages within these multi-label domains were calculated independently and were not expected to sum to 100%. Evidence maps summarized mediator families and named mediators across cardiometabolic contexts, exercise modalities, biological models, outcome domains, and evidence relations.

No meta-analysis was performed because the objective was to map a heterogeneous body of evidence spanning different study designs, exposures, biological systems, mediators, and outcomes rather than to estimate a common intervention effect. Formal risk-of-bias or certainty-of-evidence grading was not used to rank the mapped corpus. Accordingly, publication frequency and evidence-map density were interpreted only as measures of literature coverage and not as indicators of effect magnitude, causal validity, mechanistic importance, or certainty of evidence.

All final data reconstruction, harmonization, descriptive evidence synthesis, inter-reviewer agreement analyses, supplementary-table generation, and evidence-map visualizations were performed in R version 4.5.1 (R Foundation for Statistical Computing, Vienna, Austria). Details of the software environment and package versions used in the finalized workflow are provided in the **Supplementary Methods**.

### 2.8 Inter-reviewer agreement

Agreement was evaluated using the reviewers’ independent pre-adjudication decisions. For title/abstract and full-text screening, percentage agreement and Cohen’s κ were calculated for the original screening categories and for the primary versus non-primary distinction. For final charting, percentage agreement and Cohen’s κ were used for selected categorical fields. Multi-label and structural variables were additionally assessed using exact-set agreement and Jaccard similarity, where a single categorical κ would not adequately represent agreement between sets of coded values.

Agreement statistics were used as descriptive measures of the reproducibility of the screening and charting procedures and did not replace the adjudication process. The concise pre-adjudication agreement results are provided in **Supplementary Table S3**.

## 3 Results

### 3.1 Study selection and final analytic corpus

The database searches identified 3,388 records, comprising 898 from PubMed/MEDLINE, 1,099 from Web of Science Core Collection, and 1,391 from Scopus. Automated DOI- and PMID-based deduplication followed by manual resolution of residual exact-title duplicates yielded 1,535 unique records for title and abstract screening. After independent screening and adjudication, 405 reports were sought for full-text assessment; eight could not be retrieved, leaving 397 reports for full-text review. Dual full-text assessment and third-reviewer adjudication identified 383 reports that proceeded to structured full-text charting. During subsequent source-type and analytic-role adjudication, 15 reports were retained as context-only/non-primary sources and three were not retained in the final primary analytic dataset. The final primary evidence-map corpus therefore comprised 365 reports representing 363 underlying studies (**Figure 1A**). These 365 reports were published from June 2006 through August 2026.

The final relational dataset contained 413 analytically distinct exercise contrasts and 776 mediator-evidence rows. Of the 365 primary reports, 309 reports representing 308 underlying studies contributed at least one chartable named mediator. These data yielded 640 report×mediator units and 638 duplication-safe study×mediator units across 103 harmonized named mediators. Fifty-six reports representing 55 studies remained part of the primary corpus but did not contain a chartable named-mediator row and therefore did not contribute to named-mediator frequency analyses. The complete harmonized mediator vocabulary is provided in **Supplementary Table S4**, with aggregate corpus distributions summarized in **Supplementary Table S5**.

### 3.2 Study and cardiometabolic landscape

The 363 underlying studies represented a heterogeneous set of experimental and observational designs. Non-randomized controlled interventions were most frequent (145/363, 39.9%), followed by randomized parallel interventions (77/363, 21.2%), animal interventions (50/363, 13.8%), single-group interventions (50/363, 13.8%), and cross-sectional observational studies (26/363, 7.2%). The remaining studies comprised prospective observational, randomized crossover, retrospective observational, and linked multiple-report designs.

Cardiometabolic contexts were coded as non-mutually exclusive study-level attributes (**Figure 1B**). Obesity or overweight was the most frequently represented context (283/363, 78.0%), followed by insulin resistance or prediabetes (93/363, 25.6%), other coded contexts (93/363, 25.6%), endothelial or vascular phenotypes (76/363, 20.9%), type 2 diabetes (70/363, 19.3%), and diabetes not otherwise specified (55/363, 15.2%). Atherosclerosis or coronary artery disease was represented in 34 studies (9.4%), MASLD/NAFLD in 28 (7.7%), metabolic syndrome in 26 (7.2%), heart failure in 12 (3.3%), type 1 diabetes in four (1.1%), and diabetic cardiomyopathy in three (0.8%).

The breadth of these contexts was reflected in individual primary studies. Examples included a randomized intervention evaluating adiponectin and IL-6 in men with abdominal obesity (19), exercise-induced adipose Serpina3c in experimental diabetic nephropathy (20), exercise-induced Nrg4 in MASLD (21), a randomized exercise intervention in patients with metabolic syndrome (22), and aerobic exercise-induced irisin secretion in relation to endothelial function and atherosclerosis (23).

### 3.3 Mediator-family and named-mediator coverage

**Figure 2:**
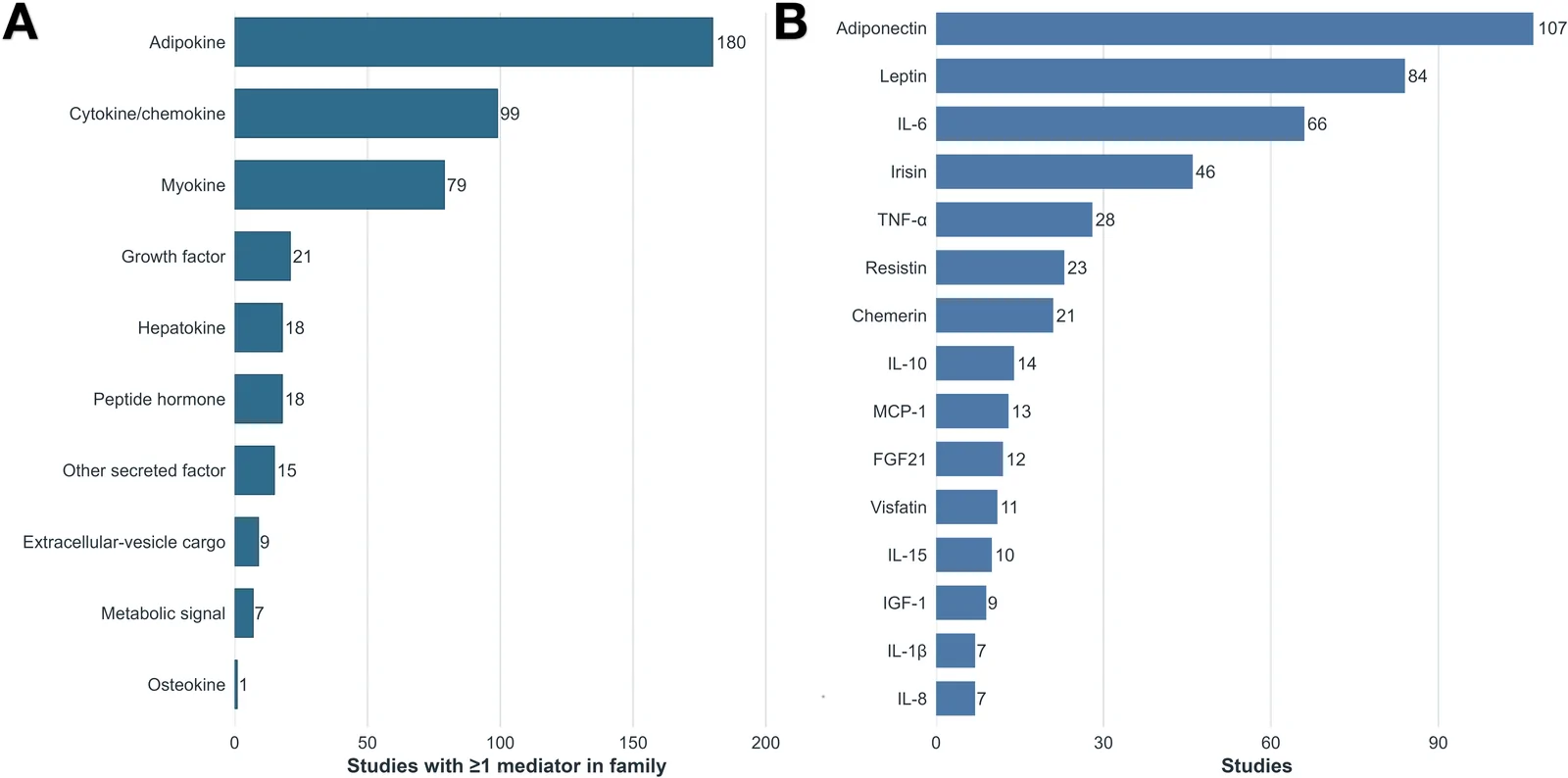
Mediator-family and recurrent named-mediator landscape. **(A)** Study-level coverage of harmonized mediator families among the 308 underlying studies contributing at least one named mediator. Individual studies could contribute to more than one mediator family but were counted at most once within a given family. **(B)** The 15 named mediators with the highest study-level coverage. The complete recurrent named-mediator distribution is shown in **Supplementary Figure S1**. Counts represent the number of underlying studies in which each mediator was mapped.

Among the 308 underlying studies contributing at least one named mediator, adipokines had the broadest study-level coverage, appearing in 180 studies (58.4%) (**Figure 2A**). Cytokines/chemokines were represented in 99 studies (32.1%) and myokines in 79 (25.6%). Other families were less frequently represented, including growth factors (21 studies), peptide hormones (18), hepatokines (18), other secreted factors (15), extracellular-vesicle cargo (9), metabolic signals (7), and osteokines (1). Because individual studies could evaluate mediators from more than one family, these counts were not mutually exclusive.

The named-mediator distribution was similarly concentrated at its upper end while retaining a substantial long tail (**Figure 2B**; **Supplementary Figure S1**). Adiponectin was mapped in 107 studies, followed by leptin in 84, IL-6 in 66, irisin in 46, TNF-α in 28, resistin in 23, and chemerin in 21. Twenty-seven of the 103 harmonized mediators were represented in at least five studies. In contrast, 56 mediators occurred in a single study and 67 occurred in no more than two studies. These counts describe the representation of mediators within the mapped corpus and were not used to rank evidence strength or biological importance.

The recurrent mediator profile was represented in both recent human interventions and experimental studies. Exercise-related adiponectin and IL-6 responses were evaluated in abdominal obesity (19), multiple endocrine responses including leptin and myostatin were examined in adolescents with obesity and leptin resistance (24), irisin was investigated in vascular and atherosclerotic models (23), and myostatin was examined in relation to exercise and insulin resistance (25). Less frequently represented signals included thymosin β4 (26), β-aminoisobutyric acid (27), N1-methylnicotinamide (28), ANGPTL4 (29), follistatin-like 1 (30), semaphorin 3C (31), and GDF15 (32, 33).

### 3.4 Exercise-exposure and family-by-context/modality landscape

Of the 413 exercise contrasts, 337 (81.6%) involved chronic training, 38 (9.2%) habitual physical activity, 33 (8.0%) an acute single bout, four (1.0%) an experimental muscle-contraction paradigm, and one (0.2%) another eligible exercise exposure. Aerobic or endurance exercise accounted for 262 contrasts (63.4%), followed by combined aerobic and resistance training (41, 9.9%), habitual physical activity (38, 9.2%), resistance exercise (37, 9.0%), and high-intensity or sprint interval training (22, 5.3%). Exercise intensity was not sufficiently reported for standardized classification in 286 contrasts (69.2%).

**Figure 3:**
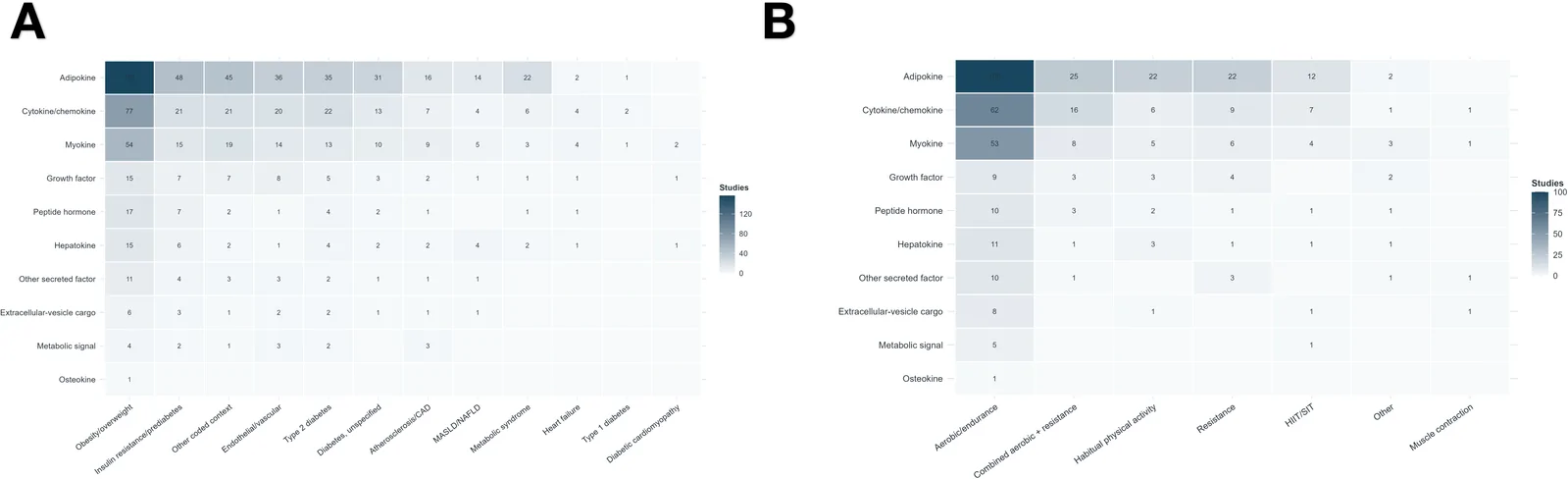
Mapping mediator families across cardiometabolic contexts and exercise modalities. **(A)** Study-level co-occurrence of mediator families and cardiometabolic contexts among the 308 underlying studies contributing at least one named mediator. Cardiometabolic contexts were coded as non-mutually exclusive attributes, and individual studies could therefore contribute to more than one context. Each underlying study was counted at most once within an individual family–context cell. **(B)** Study-level mapping of mediator families across exercise modalities using contrast-linked mediator evidence from the named-mediator-contributing study subset. Studies could contribute to more than one exercise modality when distinct eligible exercise contrasts were reported, but each underlying study was counted at most once within an individual family–modality cell. One mediator-evidence row without a direct exercise-contrast identifier was excluded from this contrast-dependent mapping.

Study-level family-by-context mapping showed that adipokine, cytokine/chemokine, and myokine studies were represented across obesity/overweight, insulin resistance/prediabetes, type 2 diabetes, endothelial/vascular phenotypes, and other coded cardiometabolic contexts (**Figure 3A**). Less frequently studied mediator families occupied fewer family–context combinations. Similarly, aerobic/endurance exercise contributed to the broadest range of mediator-family mappings, while combined aerobic–resistance training, resistance exercise, habitual physical activity, high-intensity interval exercise, and experimental contraction contributed smaller but distinct portions of the mapped evidence (**Figure 3B**).

This heterogeneity in exercise exposure was also apparent in individual studies, including objectively measured habitual physical activity in the Framingham Heart Study (34), increased physical activity with or without dietary weight loss in adults with impaired glucose metabolism (35), aerobic exercise training in people with type 2 diabetes (36), short-term exercise effects on neuronal extracellular-vesicle insulin-signaling proteins in older adults with prediabetes (37), and high-intensity interval Nordic walking in women with overweight or obesity (38).

### 3.5 Evidence-relation and biological-model architecture

**Figure 4:**
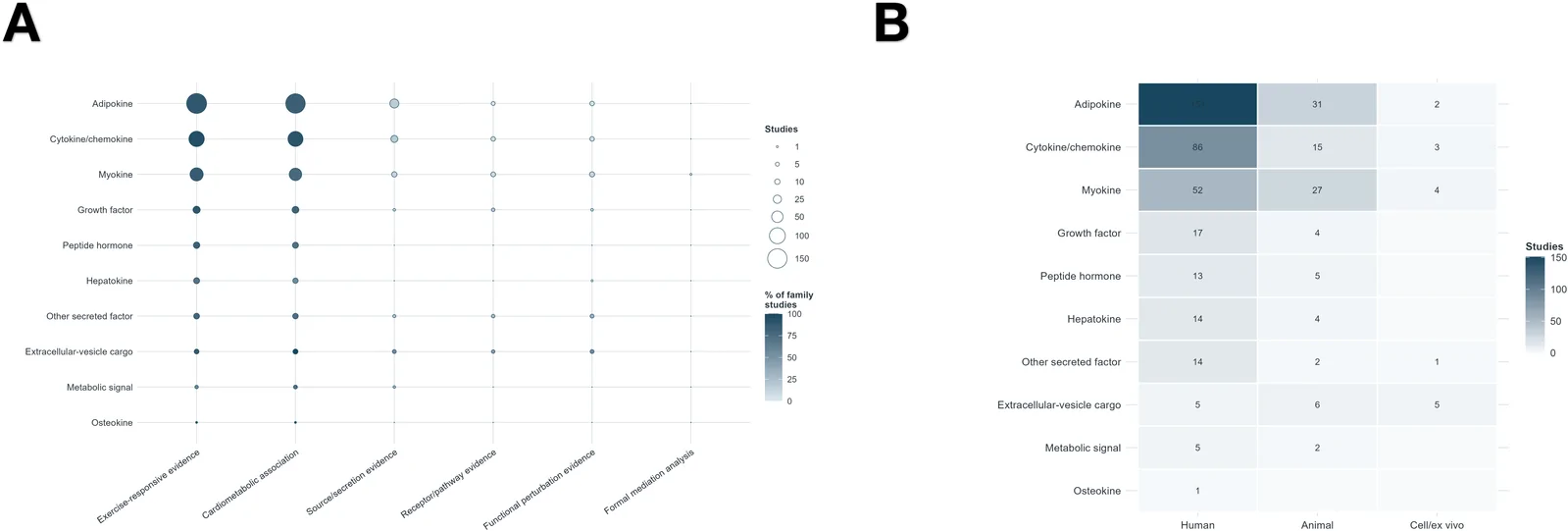
Evidence-relation and biological-model architecture of exercise-responsive endocrine-metabolic mediators. **(A)** Study-level distribution of six predefined evidence relations across mediator families among the 308 underlying studies contributing at least one named mediator: exercise-responsive evidence, cardiometabolic association, source/secretion evidence, receptor/pathway evidence, functional perturbation, and formal mediation. Evidence relations were coded independently and were non-mutually exclusive; individual studies could therefore contribute to more than one relation. Each underlying study was counted at most once within an individual family–relation cell. Formal mediation was identified in one underlying study. **(B)** Study-level co-occurrence of mediator families with human, animal, and cell/ex vivo evidence models among the 308 underlying studies contributing at least one named mediator. Biological-model categories were non-mutually exclusive because individual studies could integrate more than one biological system. Each underlying study was counted at most once within an individual family–model cell.

The six prespecified evidence relations showed markedly different levels of representation among the 308 studies contributing named mediators (**Figure 4A**). Exercise-responsive evidence was identified in 280 studies (90.9%) and cardiometabolic association in 268 (87.0%). Direct source/secretion evidence was identified in 57 studies (18.5%), receptor/pathway evidence in 30 (9.7%), functional perturbation evidence in 35 (11.4%), and formal mediation in one (0.3%). These relations were allowed to co-occur and were analyzed as distinct evidence attributes rather than as successive levels of mechanistic strength.

The smaller source/secretion, receptor/pathway, and functional-perturbation subsets nevertheless encompassed diverse experimental questions. Examples included Neuroligin-3 as an exercise-induced secreted factor linked experimentally to insulin sensitivity in obese insulin-resistant mice (39), skeletal-muscle-derived Metrnl investigated in relation to KIT/NF-κB signaling and vascular protection (40), adipose Serpina3c in exercise-related protection against diabetic nephropathy (20), and exercise-induced Nrg4 in MASLD-related cGAS–STING signaling (21). Additional studies investigated lactate/GPR81 signaling in exercise-related adipose insulin sensitivity (41), functional involvement of chemerin in exercise-related metabolic and hepatic phenotypes (42), contraction-induced GDF15 and glucose-stimulated insulin secretion (32), the FGF21–Sirtuin 3 axis in diabetic cardiomyopathy (43), and irisin–MD2 interactions in experimental NAFLD (44).

Extracellular-vesicle studies represented another set of source, pathway, and perturbational designs, including exercise-responsive exosomal let-7c-5p in atherosclerosis (45), circulating exosomal miR-214-3p in endothelial repair associated with obesity (46), post-exercise plasma extracellular vesicles with cardioprotective antioxidant activity (47), FNDC5/irisin-enriched extracellular vesicles in vascular ageing (48), muscle-derived exosomal microRNAs linked to hepatic FoxO1 and insulin sensitivity (49), and exercise-related exosomal SOD3 in type 2 diabetes (50).

Formal statistical mediation was uncommon. Only one of the 308 named-mediator-contributing studies met the prespecified requirement for an explicit mediation or indirect-effect analysis. In patients receiving maintenance hemodialysis, irisin was formally evaluated as a statistical mediator of the association between physical activity and vascular calcification (51). This classification denotes the presence of a formal statistical mediation analysis and does not by itself establish causal mediation.

Across the full primary corpus, human evidence was represented in 296 of 363 underlying studies (81.5%), animal evidence in 163 (44.9%), and cell or ex vivo evidence in 97 (26.7%). These biological-model categories were non-mutually exclusive because individual studies could integrate more than one biological system. Among the 308 studies contributing at least one named mediator, the distribution of human, animal, and cell/ex vivo evidence across mediator families is shown in **Figure 4B**. Cross-model examples included serum proteomic identification and subsequent evaluation of CD300LG (52) and organism-wide cell-type-resolved secretome mapping across multiple tissues (53). The overlapping model categories were retained separately rather than collapsed into a composite evidence score.

Mediator-family linkage to prespecified outcome domains and the distribution of reported mediator-response directions are shown in **Supplementary Figures S2** and **S3**, respectively.

### 3.6 Inter-reviewer agreement

Pre-adjudication agreement was assessed separately for study selection and final *de novo* data charting (**Supplementary Table S3**). At title and abstract screening, agreement was 79.5% with Cohen’s κ = 0.683 for the original three-category decision and 92.3% with κ = 0.804 for the primary versus non-primary distinction. At full-text assessment, corresponding agreement was 94.5% with κ = 0.307 for the three-category decision and 94.7% with κ = 0.338 for the primary versus non-primary distinction.

For final data charting, agreement was evaluated before adjudication after both reviewers had independently re-charted the 383 reports. Among 272 mediator units that could be matched between the independent reviewer datasets, mediator-family classification showed 90.8% agreement (κ = 0.868) and evidence-model classification showed 86.8% agreement (κ = 0.652). For variables represented as sets rather than single categories, cardiometabolic-context sets showed 23.0% exact-set agreement with a mean Jaccard similarity of 0.583, and evidence-relation sets showed 36.4% exact-set agreement with a mean Jaccard similarity of 0.533. At the report level, reviewers agreed on the number of exercise-contrast rows for 86.7% of reports; complete structured exercise-contrast signatures showed 54.6% exact-set agreement with a mean Jaccard similarity of 0.553. All final analytic values were derived from the reconciled and adjudicated dataset.

## 4 Discussion

### 4.1 Principal findings

This systematic scoping review and evidence map shows that research on exercise-responsive endocrine-metabolic mediators has developed into a large but unevenly distributed literature. Three findings are particularly relevant. First, the evidence base was concentrated in obesity/overweight and related metabolic phenotypes and was dominated by chronic training and aerobic/endurance exercise. Second, a relatively small group of mediators, particularly adiponectin, leptin, IL-6, and irisin, accounted for a substantial proportion of study coverage, alongside a much larger set of infrequently studied signals. Third, the types of evidence reported were markedly uneven: exercise-responsive changes and cardiometabolic associations were common, whereas direct investigation of source/secretion, receptor/pathway involvement, functional perturbation, and especially formal mediation was substantially less frequent.

These findings complement previous systematic reviews that have estimated exercise effects on selected myokines or adipokines in defined populations and exercise settings (12–15, 54, 55). Such syntheses are well suited to questions concerning the magnitude and direction of changes in particular biomarkers. Our review addresses a different question by mapping how the broader primary literature is distributed across mediators, cardiometabolic contexts, exercise exposures, biological models, and evidence relations. This distinction is important because the frequency with which a mediator has been studied does not indicate the strength, consistency, or causal importance of its biological role.

### 4.2 Exercise responsiveness and mechanistic attribution are distinct questions

The clearest structural feature of the evidence map was the predominance of studies documenting exercise-responsive changes or cardiometabolic associations over studies directly investigating biological source or downstream function. This distinction has practical implications for how the exerkine literature is interpreted. A circulating mediator may change after exercise and correlate with improved glucose metabolism, adiposity, or vascular function without the study establishing which tissue produced that change, whether the mediator reached a biologically relevant target, or whether altering the mediator modifies the exercise response.

Several studies in the mapped corpus illustrate how these questions can be addressed more directly. Exercise-induced Metrnl was investigated together with endothelial signaling and functional manipulation (40), adipose Serpina3c was examined in experimental diabetic nephropathy (20), and exercise-induced Nrg4 was linked experimentally to hepatic signaling in MASLD (21). Particularly detailed examples included the FGF21–SIRT3 axis in diabetic cardiomyopathy, in which genetic deletion of FGF21 or its cardiac co-receptor altered the cardioprotective response to exercise (43), and the demonstration that exercise-induced irisin can interact directly with MD2 in experimental NAFLD (44). Such designs provide information beyond a change in circulating concentration by directly addressing biological source, target, pathway involvement, or functional contribution.

This distinction is increasingly emphasized in contemporary discussions of exercise biology, which describe exerkines within networks of interorgan signaling rather than as isolated circulating biomarkers (2–4, 56–58). Studies directly investigating these source-, pathway-, or function-related questions nevertheless represented a minority of the mapped literature. This should not be interpreted as an ordinal hierarchy in which association studies are intrinsically uninformative or perturbational studies automatically establish clinical relevance. Rather, these study designs address different biological questions and become particularly informative when findings can be connected across complementary forms of evidence.

The same caution applies to mediation. Functional perturbation and formal statistical mediation are related but non-equivalent approaches. An intervention in which knockout, blockade, supplementation, or transfer modifies an exercise-related phenotype provides experimental evidence of functional involvement but is not a statistical mediation analysis. Conversely, an exercise-associated change in a mediator that correlates with an outcome does not establish an indirect effect. Only one study in the present corpus met our prespecified definition of formal mediation (51). The rarity of formal mediation in the mapped literature suggests that this analytical approach has seldom been applied to exercise–mediator–outcome relationships. Where appropriate, future longitudinal human studies could use temporally ordered mediation designs to complement, rather than replace, experimental evidence.

### 4.3 A concentrated literature within an expanding molecular landscape

Adiponectin and leptin were the two most frequently represented named mediators, and adipokines were the largest mediator family. This pattern is consistent with the high representation of obesity and overweight in the mapped corpus and with the longstanding use of adipokines as markers of insulin resistance, adiposity, and systemic inflammation. Recent quantitative syntheses continue to show exercise-related changes in adiponectin and leptin that vary according to exercise modality and dose in populations with overweight, obesity, and type 2 diabetes (54, 55).

High study frequency, however, is partly a property of the history and accessibility of a research question. Mediators that entered clinical biomarker research earlier, can be measured readily in blood, and are relevant to prevalent metabolic conditions have had more opportunity to accumulate publications. The numerical predominance of adiponectin, leptin, IL-6, or irisin should therefore not be interpreted as evidence that these molecules necessarily account for a greater proportion of the systemic benefits of exercise than less frequently investigated signals.

At the same time, the candidate landscape is expanding. Multi-tissue and multi-omic studies have demonstrated that exercise produces coordinated molecular responses extending well beyond a small set of circulating proteins (9, 10, 56). Within our primary corpus, proteomic and secretome approaches identified exercise-responsive proteins such as CD300LG and numerous cell-type-specific secreted factors (52, 53), while extracellular-vesicle studies extended the mapped evidence to proteins and non-coding RNAs transported between cells (45–50). Recent methodological and biological work on extracellular vesicles also highlights an important interpretive issue: changes in circulating vesicles or their cargo do not automatically identify their tissue of origin or demonstrate delivery to a specific target tissue (59, 60).

These observations support a shift from simply expanding lists of candidate exerkines toward establishing traceable biological relationships. For a newly identified mediator, informative follow-up studies may therefore include replication across exercise conditions, characterization of temporal responses, evidence concerning cellular or tissue origin, identification of biologically plausible target tissues, and experimental testing of downstream function. The value of such work lies in connecting complementary observations rather than in assigning a universal ranking to individual mediators.

### 4.4 Exercise exposure is part of the biological signal

A second important finding was the marked concentration of the literature in chronic training and aerobic/endurance exercise. More than four-fifths of mapped exercise contrasts involved chronic training, whereas acute single-bout exercise, habitual physical activity, and experimental contraction were less frequently represented. Across exercise modalities, aerobic/endurance exercise predominated, while resistance exercise, combined aerobic–resistance training, and high-intensity or sprint interval training accounted for smaller portions of the mapped evidence. This distribution matters because an exercise-responsive mediator is not a fixed property independent of the exercise stimulus. Acute and chronic exercise can generate different molecular profiles, and responses may vary with modality, intensity, duration, training status, and sampling time (11, 14, 15, 56).

Recent meta-analytic evidence reinforces this point. Exercise modality and dose appear to influence adipokine responses in overweight and obesity (54), while a 2026 network and dose-response meta-analysis in type 2 diabetes identified modality-specific patterns in several commonly studied exerkines in addition to differences in glycemic outcomes (55). These findings help explain why apparently inconsistent mediator responses across individual studies need not represent biological contradiction; they may instead reflect differences in exercise prescription, study population, or measurement timing.

Against this background, the finding that exercise intensity could not be standardized for 69.2% of the mapped contrasts is consequential. Incomplete reporting of exercise interventions is a broader problem in the literature. The Consensus on Exercise Reporting Template was developed specifically to improve the reproducibility of exercise interventions (61), and subsequent evaluations have shown incomplete reporting both across clinical exercise research generally and in trials involving type 2 diabetes (62, 63). For molecular exercise studies, reporting requirements extend beyond the conventional frequency, intensity, time, and type of exercise. The interval between the final exercise bout and biospecimen collection, fasting status, time of day, sampled biological compartment, and relevant pre-analytical procedures can also materially influence interpretation of an exercise-responsive signal.

More complete reporting would therefore improve both reproducibility and biological interpretation. It would also permit future evidence syntheses to move beyond broad modality categories toward more informative analyses of dose, temporal response, and interactions with participant characteristics.

### 4.5 Implications for the next generation of exercise-mediator studies

The present map suggests that further progress will depend less on measuring an ever-larger number of circulating factors in isolation and more on linking discovery with experimental and clinical validation. High-dimensional profiling remains valuable for identifying candidate signals and coordinated molecular programs, particularly as multi-tissue datasets provide increasingly detailed maps of exercise adaptation (10, 56). However, discovery and mechanistic attribution should be treated as distinct but connected questions.

One useful strategy is to combine well-characterized exercise interventions with time-resolved blood sampling and, where feasible, tissue-level measurements. Candidate mediators emerging from these studies can then be evaluated through source/secretion experiments, receptor or pathway interrogation, or functional perturbation in appropriate experimental systems. Several studies in the present corpus already demonstrate the value of such complementary designs (20, 21, 39, 40, 43, 44, 48, 49). Extracellular-vesicle studies provide a particularly relevant example because cargo discovery, tissue attribution, target-cell uptake, and functional testing are distinct experimental questions (59, 60).

For human studies, stronger temporal designs could also support more informative mediation analyses. Ideally, the exercise exposure should precede the mediator measurement, which in turn should precede the downstream outcome, with sufficient attention to confounding and measurement error. Formal mediation could then complement, rather than substitute for, experimental evidence. Similar caution is warranted as exerkines are increasingly discussed as therapeutic targets or exercise mimetics in cardiometabolic disease (4, 57, 58). The therapeutic potential of an exercise-responsive mediator ultimately requires evidence that extends beyond its association with exercise and includes reproducible biological activity, appropriate target engagement, and relevance to human disease.

These priorities do not require every study to span the entire pathway from exercise exposure to clinical outcome. Population studies, controlled interventions, omics discovery, tissue-level experiments, and functional models each make different contributions. Greater progress may instead come from designing these studies so that their findings can be connected across a common evidence framework.

### 4.6 Limitations

First, although the search strategy was implemented across PubMed/MEDLINE, Web of Science Core Collection, and Scopus using database-specific controlled vocabulary and free-text terminology, additional specialized bibliographic databases and trial registries were not searched. Eligible studies indexed only in sources outside the three databases searched may therefore have been missed, and variation in indexing and terminology means that complete retrieval of every potentially eligible report cannot be guaranteed. A formal independent PRESS review, a formal sentinel-study validation exercise, and exhaustive backward and forward citation searching of every included report were not undertaken. The primary evidence map was also restricted to studies for which an English-language full text was available. These considerations should be borne in mind when interpreting the mapped corpus as a structured representation of the literature identified by the review methods rather than as an exhaustive census of every potentially eligible publication.

Second, the included literature was highly heterogeneous with respect to study design, population, exercise exposure, biological model, sampling schedule, biospecimen, assay, mediator, and outcome. A single pooled effect estimate would therefore not have represented the objective or structure of this review. Formal study-level risk-of-bias assessment or certainty-of-evidence grading was not undertaken because the purpose was descriptive evidence mapping rather than estimation of a common intervention effect or ranking of individual mediators. Accordingly, study frequency and map density should not be interpreted as measures of effect magnitude, causal validity, mechanistic importance, or certainty of evidence. Focused systematic reviews addressing specific mediators or clinical questions may require design-specific risk-of-bias assessment and quantitative synthesis.

Third, limitations in primary-study reporting constrained several harmonized variables, most notably exercise intensity, which could not be standardized for 69.2% of exercise contrasts. Biological source can also be difficult to establish for circulating factors produced by multiple tissues, and the present classifications necessarily depended on what individual studies directly investigated and reported. Finally, pre-adjudication agreement was lower for some granular and multi-label charting domains than for simpler categorical classifications. These discrepancies were resolved through the prespecified reconciliation and adjudication process but illustrate the interpretive complexity of mapping a heterogeneous molecular literature.

## 5 Conclusion

The literature on exercise-responsive endocrine-metabolic mediators in diabetes and cardiometabolic disorders is extensive but unevenly distributed. Research was concentrated in chronic training and aerobic/endurance exercise, obesity-related contexts, and a relatively small group of recurrent mediators, while the molecular landscape continues to expand through multi-omic, secretome, and extracellular-vesicle approaches. Exercise-responsive changes and cardiometabolic associations were frequently represented, whereas direct investigation of biological source, downstream signaling, functional contribution, and formal mediation was less common. Distinguishing these evidence relations provides a clearer framework for interpreting the current literature and highlights opportunities for future studies to connect well-characterized exercise exposures with temporal mediator profiling, source validation, pathway interrogation, and functional testing.

## Data Availability

The original contributions presented in this study are included in the article and **Supplementary Material**. Additional derived data and analysis code supporting the reported results are available from the corresponding authors upon reasonable request, subject to applicable database-licensing and third-party copyright restrictions.
