# Supplementary material for: Exercise-responsive endocrine-metabolic mediators in diabetes and cardiometabolic disorders: a systematic scoping review and evidence map
Source: Front Endocrinol (Lausanne). 2026 Sep 22;17:1892959. doi: 10.3389/fendo.2026.1892959 (PMC13602954; doi:10.3389/fendo.2026.1892959)
Supplement: Supplementary file 1 [file SupplementaryFile1.docx]

Supplementary Material

# 1 Supplementary Methods

## 1.1 Search strategy and record processing

PubMed/MEDLINE, Web of Science Core Collection (WoSCC), and Scopus were searched from database inception through 7 August 2026, with all final searches conducted on 7 August 2026. The final strategies used a common three-part conceptual structure comprising exercise-responsive mediators, cardiometabolic disease or risk contexts, and a broad molecular/biological relevance component incorporating mechanism-, biomarker-, signaling-, and omics-related terminology together with terms for inflammation, oxidative stress, mitochondrial biology, and endothelial function. PubMed/MEDLINE used Medical Subject Headings and title/abstract terms, WoSCC used Topic fields, and Scopus used TITLE-ABS-KEY. No language, publication-type, document-type, or journal-source filters were applied within the database queries. The complete executed strategies are provided in Supplementary Table S1.

The searches yielded 898 records from PubMed/MEDLINE, 1,099 from WoSCC, and 1,391 from Scopus, giving 3,388 records before deduplication. Records were standardized before sequential deduplication. DOI-based deduplication removed 1,815 records, followed by 29 additional removals using PMID information, leaving 1,544 records after automated deduplication. Seventeen residual exact-title candidate pairs were then reviewed manually, resulting in nine additional merges and a final corpus of 1,535 unique records for title/abstract screening.

## 1.2 Screening and full-text eligibility assessment

The 1,535 deduplicated records were independently screened by two reviewers using the predefined eligibility criteria. Disagreements relevant to the primary evidence stream were resolved through third-reviewer adjudication. Following this process, 405 reports were sought for full-text retrieval.

Eight reports could not be retrieved, leaving 397 reports for independent full-text assessment. Eligibility decisions were based on the full article rather than bibliographic metadata or abstract text alone. Following independent full-text assessment and third-reviewer adjudication, 383 reports proceeded to structured full-text charting; 13 reports were excluded at the full-text stage and one secondary contextual synthesis was retained outside the primary evidence stream.

Primary research and contextual or non-primary evidence were maintained separately. During structured charting, source type, exercise anchoring, and analytic role were verified from the full text before the final primary evidence-map dataset was established.

## 1.3 Data-charting framework

Data charting was organized as a relational structure comprising three linked analytic levels: Report Level, Exercise Contrast, and Mediator Evidence. Report-level variables described study design, biological model, population or experimental condition, sample-size information, sex composition, cardiometabolic contexts, and information relevant to companion-report identification. Exercise-contrast variables captured exercise temporality, modality, intensity, comparator structure, programme characteristics, and co-interventions. Mediator-evidence variables captured the reported mediator, mediator family, biological evidence model, measurement method, biospecimen, sampling time, response direction, cardiometabolic outcome domains, and predefined evidence relations.

The initial full-corpus charting exercise was treated as diagnostic. Operational definitions were subsequently refined in a targeted 20-report pilot. After these rules were finalized, two new blank reviewer workbooks were generated for the complete 383-report corpus, and both reviewers independently re-charted all reports de novo from the full text. Previous diagnostic extraction workbooks were not used as extraction inputs for this final charting round. Formal charting used information from the main text, Methods, Results, tables, figures, and supplementary material where available.

## 1.4 Multi-label coding, harmonization, and companion reports

Cardiometabolic contexts and outcome domains were coded as non-mutually exclusive variables. A report or underlying study could therefore contribute to more than one applicable context or outcome domain. Distinct exercise exposures or comparators were represented as separate exercise contrasts when they yielded independently interpretable results.

Mediator terminology was initially recorded in source-faithful form. Synonymous or equivalent mediator names were harmonized centrally after independent charting and adjudication, and mediator-family assignments were finalized at the same stage. This preserved the terminology used in individual reports while providing a consistent vocabulary for study-level synthesis.

Potential companion publications were evaluated using available trial or cohort identifiers, population characteristics, recruitment periods, protocols, intervention characteristics, and other study-level information. Reports judged to arise from the same underlying study were assigned a shared study identifier before study-level aggregation.

## 1.5 Evidence-relation definitions

Six evidence relations were coded independently.

Exercise-responsive evidence indicated a directly reported change in, or association of, the mediator with exercise, physical activity, or experimental contraction.

Cardiometabolic association indicated an explicit within-study relationship between the mediator and an eligible cardiometabolic phenotype or outcome; membership in a disease population alone was not sufficient.

Source/secretion evidence required direct evidence supporting production, release, secretion, or tissue/cellular origin of the mediator. Tissue expression without evidence of release or source attribution was not sufficient.

Receptor/pathway evidence required mediator-specific evidence linking the mediator to a receptor or downstream biological pathway.

Functional perturbation evidence required direct manipulation of the mediator, its receptor, or a mediator-linked pathway together with assessment of a downstream biological or cardiometabolic effect.

Formal mediation required an explicit statistical mediation or indirect-effect analysis testing the mediator as an intermediate variable between exercise or physical-activity exposure and a cardiometabolic outcome.

The six relations were non-mutually exclusive and were not combined into a composite evidence hierarchy.

## 1.6 Inter-reviewer agreement and adjudication

Pre-adjudication agreement was quantified separately for title/abstract screening, full-text eligibility assessment, and selected structured data-charting domains. Percentage agreement and Cohen's κ were used for single-label categorical decisions. Exact-set agreement and Jaccard similarity were used for multi-label or structured domains for which a single categorical κ would not adequately describe agreement.

Agreement statistics were calculated from the reviewers' independent decisions before reconciliation. Discordant screening and charting decisions were subsequently resolved through reviewer reconciliation or third-reviewer adjudication before the analytic dataset was finalized. Selected pre-adjudication agreement statistics are provided in Supplementary Table S3.

## 1.7 Analytic units and evidence-map construction

Distinct analytic units were retained throughout synthesis. Publication-level variables were retained at the report level, whereas companion reports were linked before duplication-safe study-level summaries. Exercise characteristics were summarized using analytically distinct exercise contrasts, and mediator-specific analyses used mediator-evidence and study×mediator representations according to the question being addressed.

The final primary dataset contained 365 primary reports representing 363 underlying studies, 413 exercise contrasts, 776 mediator-evidence rows, 640 report×mediator units, and 638 study×mediator units. A total of 103 harmonized named mediators were represented, with 308 underlying studies contributing at least one named mediator. One mediator-evidence row lacked a direct exercise-contrast identifier and was retained in analyses for which contrast linkage was not required but excluded from contrast-dependent mappings.

For mediator and mediator-family summaries, each underlying study contributed at most once to a given mediator or family. In multi-label cross-tabulations, studies could contribute to multiple applicable categories but were counted at most once within an individual matrix cell. Counts and percentages were used to describe the distribution of the mapped literature; no pooled treatment-effect estimate or composite evidence score was calculated.

## 1.8 Software environment and computational reproducibility

The finalized computational workflow was executed in R version 4.5.1 (2025-06-13 ucrt) on a Windows 11 x64 platform (x86_64-w64-mingw32). The principal analytical and visualization packages used in the finalized workflow were cowplot 1.2.0, data.table 1.18.4, dplyr 1.2.1, forcats 1.0.1, ggplot2 4.0.3, magick 2.9.1, openxlsx 4.2.8.1, purrr 1.2.2, readr 2.2.0, readxl 1.5.0, stringdist 0.9.17, stringr 1.6.0, tibble 3.3.1, and tidyr 1.3.2. Workflow-support packages included rstudioapi 0.18.0 and zip 3.0.2. Versioned session information was retained for the final data-reconstruction, evidence-synthesis, figure-generation, inter-reviewer-agreement, and supplementary-table stages. No stochastic modelling or dimensionality-reduction analysis was retained in the final analytical workflow; consequently, the reported final analyses did not depend on random-seed initialization

# 2 Supplementary Figures

## 2.1 Supplementary Figure S1

**Supplementary Figure S1. Recurrent named-mediator landscape in the primary evidence map.**
Study-level coverage of recurrent harmonized named mediators among the 308 underlying studies contributing at least one named mediator. Mediators represented in at least five underlying studies are displayed; this threshold was used only to improve visual readability and does not indicate evidence strength, biological importance, or certainty. Each underlying study was counted at most once for an individual named mediator.

## 2.2 Supplementary Figure S2

**Supplementary Figure S2. Mapping mediator families across cardiometabolic outcome domains.**
Study-level co-occurrence of mediator families and prespecified cardiometabolic outcome domains among studies contributing named mediators. Outcome domains were coded as non-mutually exclusive attributes, and individual studies could therefore contribute to more than one outcome domain and more than one mediator family. Each underlying study was counted at most once within an individual family–outcome cell. The map describes the distribution of reported outcome linkages and should not be interpreted as evidence of causal effects.

## 2.3 Supplementary Figure S3

**Supplementary Figure S3. Direction-of-response landscape across named mediators.**
Distribution of reported response directions across the 638 duplication-safe study×mediator units. When multiple mediator-evidence rows contributed to the same study×mediator unit, they were collapsed at the unit level with mixed directional states retained where applicable. Direction categories therefore describe the distribution of reported findings within the mapped corpus and should not be interpreted as pooled effect directions, effect magnitudes, or evidence strength.

# 3 Supplementary Tables

Supplementary Tables S1–S5 are provided in the accompanying Excel file.
